# Flipping the Dental Anatomy Classroom

**DOI:** 10.3390/dj6030023

**Published:** 2018-06-21

**Authors:** Sergio Varela Kellesarian

**Affiliations:** Department of General Dentistry, Eastman Institute for Oral Health, University of Rochester, Rochester, NY 14620, USA; sergio_kellesarian@urmc.rochester.edu; Tel.: +1-786-717-0150

**Keywords:** dental anatomy, flipped classroom, education, dental morphology

## Abstract

The development of cognitive knowledge, motor skills, and artistic sense in order to restore lost tooth structure is fundamental for dental professionals. The course of dental anatomy is taught in the initial years of dental school, and is a component of the basic core sciences program in the faculties of dentistry. The learning objectives of the dental anatomy course include identifying anatomical and morphological characteristics of human primary and permanent teeth; identifying and reproducing tooth surface details in order to recognize and diagnose anatomical changes; and developing student’s psychomotor skills for restoring teeth with proper form and function. The majority of dental schools rely on traditional methods to teach dental anatomy, using lectures to convey the theoretical component; whereas the practical component uses two-dimensional drawing of teeth, identification of anatomical features in samples of preserved teeth, and carving of teeth. The aim of the present literature review is to summarize different educational strategies proposed or implemented to challenge the traditional approaches of teaching dental anatomy, specifically the flipped classroom educational model. The goal is to promote this approach as a promising strategy to teaching dental anatomy, in order to foster active learning, critical thinking, and engagement among dental students.

## 1. Introduction

Knowledge of tooth morphology and dental anatomy and function is imperative for the practice of modern dentistry. Dental professionals must develop cognitive knowledge, motor skills (manual dexterity), and artistic sense, in order to restore the lost tooth structure with different materials [1,2]. These abilities are fundamental in operative dentistry, aesthetics, and oral rehabilitation, where the main objectives are to mimic dental restorations to natural teeth, return functionality and prevent overloading [1,3]. Likewise, knowledge of dental anatomy and tooth morphology is fundamental in other specialties including oral surgery, endodontics, forensics, periodontics, and prosthodontics [3,4].

Dental anatomy is one of the first courses taught in dental school, and is a component of the basic core sciences program in the faculties of dentistry around the world. The learning objectives of the dental anatomy course include identifying anatomical and morphological characteristics of human primary and permanent teeth; identifying and reproducing tooth surface details in order to recognize and diagnose anatomical changes [1]; and developing students’ psychomotor skills for restoring teeth with proper form and function [5]. Commonly the dental anatomy course includes a theoretical and a practical component. Most dental schools continue to use traditional methods of dental anatomy teaching, relying on lectures (faculty member showing slides and describing the anatomical features of each tooth) for the theoretical component. In contrast, the practical component, is designed to develop students’ psychomotor skills and uses two-dimensional drawings of teeth, identification of anatomical features in samples of preserved teeth, and carving of teeth. Usually the carving exercises are completed in oversized wax and/or soap blocks [5,6]. These materials are used due to relative low cost, easy handling, and good reproducibility [1].

Although these traditional techniques have been used to teach dental anatomy for decades in dental schools in the U.S. and many countries around the world, several concerns have been raised about their efficacy. One of the main limitations of the dental anatomy course is that it is usually isolated from other courses related to patient care, and present poor emphasis to clinical relevance [1]. Likewise, the course usually fails to prepare students to transition from pre-patient care to real clinical scenarios [5]. Moreover, the traditional lecture model presents several limitations, including one-way communication, lack of interaction, and poor student engagement [7]. Studying the theory of dental anatomy alone is not enough to acquaint students with the anatomy of each tooth in detail. Similarly, using the gold-standard oversized wax/soap blocks carving has been also criticized. Dental students should develop an ability to analyze volume, form, and function of each tooth and restore both the aesthetics and the complete physiology. However, using models with oversized dimensions impair the perception of proper proportion [2]. As a result, neither psychomotor nor cognitive skills are learned in the context of clinical practice.

The aim of the present literature review is to summarize different educational strategies proposed or implemented to challenge the traditional approaches of teaching dental anatomy, specifically the flipped classroom educational model. The goal is to promote this approach as a promising strategy to teaching dental anatomy, in order to foster active learning, critical thinking, and engagement among dental students.

## 2. Challenging the Traditional Teaching of Dental Anatomy

In recent years, dental schools around the world have proposed innovative educational strategies and alternatives to the current curricula, challenging the traditional teaching of dental anatomy. Kieser et al. incorporated spontaneous storytelling with problem-based learning, in their dental anatomy course in the University of Otago, New Zealand, in order to stimulate deep learning in a dental anatomy class [8]. The use of stories has been proposed as a tool for storing and describing experiential knowledge. During storytelling, the stories act as examples of concepts, principles, and theories presented during instruction. Studies have showed that students are more receptive to learning through listening to stories of how others had confronted similar problems [9]. In this context, findings from Kieser et al. showed significantly higher levels of satisfaction in the group of students exposed to stories (93.1%) compared with the group without histories (55.8%). They suggested that providing a narrative plays a role in the comprehension and organization of events; concluding that storytelling is a meaningful tool to foster satisfaction and reflective learning during dental anatomy teaching [8].

New technologies have been designed and implemented to modify traditional aspects of dental anatomy teaching. These technologies include the use of interactive software, three-dimensional (3D) images, and digital atlases to study detailed tooth morphology, virtual patient simulations, and electronic flashcards [10]; likewise, digital video discs (DVDs) and YouTube videos have become popular tools to teach wax carving techniques [1,2]. Bogacki et al. used equivalence testing to show that teaching of dental anatomy with an interactive computer program was found to be as effective as teaching with traditional lectures [11]. Whereas, Al-Thobity et al. reported that students who received software-facilitated teaching performed better than the students who did not receive it [7]. Similarly, Nance et al. evaluated the efficacy of a DVD demonstrating wax carving compared with traditional techniques. No significant difference in terms of student’s grades was reported; however, students in the DVD group reported a higher favorable perception, compared with the traditional approach [12]. Moreover, virtual reality simulation and haptic technology are emerging as new innovations to minimize the gap between pre-clinical and clinical courses in dental anatomy [13,14,15]. In a recent study conducted by Bakr et al., 24 dental students were trained using a virtual reality dental simulator. The results suggested that although haptic simulators could not replace traditional training, it might be used as a valuable supplementary tool to develop preclinical dental skills [16].

Teaching with digital tools has shown promising results by stimulating interactivity and independence in the learning experience [1,17,18]. They are considered to be useful instruments for effectively representing and integrating the spatial and symbolic domains of anatomical information [6,19,20]. Moreover, digital media as an adjunctive tool offers excellent visualization and allows students to have access to the information any time, to review the material several times as needed and allow for flexible hours for studying [2,6,7]. Furthermore, these programs are easy to use and provide self-assessment tools to further aid students [7].

### 2.1. Flipped Classroom

A flipped classroom (also known as the backwards or inverse classroom) is an instructional strategy and a type of blended learning methodology that reverses the traditional learning environment, combining online learning, and the face-to-face classroom. This methodology leverages computer technology to reverse the traditional learning environment, delivering instructional contents outside the classroom, allowing students to acquire basic knowledge and concepts through the use of audiovisual tools (such as short videos, instructor’s prerecorded lectures, etc.) hosted online [21,22]. The students watch these videos on their own schedule before class time, resulting in self-guided and self-paced learning [23]. The goal is to shift the learning from being instructor-centered to student-centered; each student is responsible for presenting to the class a basic understanding of key concepts, in order to allow them to engage and participate in class discussion and problem-solving activities [24,25]. The fundamental idea is to is create a flexible working environment during the face-to-face sessions, moving the lower levels of Blooms taxonomy (remember, understand) outside the group learning space; in this way, the students will apply the higher levels (create, evaluate, analyze, and apply) of Blooms Taxonomy to the tasks and skills under the facilitation and interaction with the instructor [26].

### 2.2. Flipped Classroom in Dentistry

The flipped classroom has been introduced successfully in the past two decades in different fields of dental education in order to promote critical thinking, problem-solving, and self-directed assessment among dental students [27]. Faraone et al. provided online audio-visual and written support material for theoretical and laboratory procedures prior to students taking a complete dental course. Their findings suggested favorable outcomes, including high levels of performance and satisfaction among students [28]. Similarly, Nishigawa et al. compared the effects of flipped classroom and team-based learning (TBL) in fixed prosthodontics education. The results showed that there was no significant difference in the examination score between flipped classroom and TBL, concluding that both styles are valid formats for clinical education compared to traditional lecture styles [22]. Likewise, other studies have suggested the validity of this educational method in pediatric dentistry [27,29], orthodontics [30], prosthodontics [31], oral radiology [32], anatomy and neuroanatomy [33], and biodiversity [34]. The flipped classroom has the potential to increase dental students’ motivation and improve their perception of the quality of the learning experience.

### 2.3. Flipping the Dental Anatomy Classroom

The flipped classroom approach combined with online learning tools have been successfully used to increase student engagement and support student learning in the dental anatomy program [2,3,5,26,35,36,37]. Obrez et al. from the University of Illinois at Chicago, proposed for the first time in indexed literature to revise the dental anatomy curriculum, in order to integrate independent class preparation with active small-group discussion and patient scenario-based wax-up scenario (using mannequins instead of oversized wax-blocks) to replace missing tooth structure [5]. Bakr et al. implemented a partial flipped classroom model in Griffith University, Queensland, Australia; including a series of online videos, access to an online digital library, and online quizzes [37]. Similarly, Abu Eid et al. reported changes introduced in the dental anatomy course in the University of Aberdeen Dental School, Scotland. The course changed the traditional approaches, and now is delivered through online handouts that students discuss during designated self-directed workshops facilitated by an instructor. The workshops include using different tools such as plastic and natural teeth; the tooth carving is now completed in regular sized models; and clinical implications of defective dental morphology are discussed [3]. Similar curricular changes have been completed in Harvard School of Dental Medicine at Boston [35,36]; and University of Glasgow, Scotland [26]. The main goals of this new flipped classroom approach include: using class time to engage students in small-groups discussion and practice; delivering the majority of foundational content outside the class using technological tools or instructor-provided digital learning resources; and facilitating student transition from pre-patient care to clinical practice [5].

### 2.4. Evidence-Based Recommendations

The implementation of a flipped dental anatomy classroom was proposed in order to enhance student’s cognitive and psychomotor skills in human tooth morphology. The dental anatomy course might be offered in the same traditional time (half-day per week) in the first year of dental school. The traditional lecture approach will be substituted by a flipped classroom, incorporating didactic activities and a laboratory component [36].

Students will be required to study the theoretical and foundational knowledge before each class. Content will be delivered using an on-line blackboard; different technological tools might be used, including but not limited to syllabus, course manual (developed by the instructor), instructional videos, short PowerPoint slide presentations (15–30 min voiceover slides with annotations), clinical cases, access to 3D software, dental atlases, and others. A low stakes quiz (5–10 multiple choice questions) addressing the first two levels of Blooms taxonomy, will be completed on-line before the class encounter, in order to ensure that students had reviewed the online material [3,5,35]. The instructor will begin the class by setting the main goals, expected learning outcomes, and outlining the activities planned for the face-to-face session. The presentation will be followed by small-group discussions (clinical cases) and simulation (laboratory exercises). The instructor will act as a facilitator, encouraging discussion, critical-thinking, and understanding of clinical rationale of the different activities. The traditional carving of over-sized wax or soap blocks will be replaced by the incorporation of simulation activities with manikin teeth and typodonts. These teeth will be normal-sized; replicating typical cavities preparations in anterior and posterior teeth surfaces. For example, the molar preparations will require restoration of the marginal ridges, contact points, occlusal and interproximal surfaces, and pits and fissures. The students will develop psychomotor (visual and motor) skills by waxing missing teeth structure and re-establishing proper morphology [5,31,35,36].

Student learning/performance will be assessed during each class session. The evaluation will include instructor and peer assessments. Instructor assessment will evaluate students’ attendance to class sessions, active participation in the small-groups discussion, completion of all the laboratory work, and a performance examination using standardized assessment tools. Likewise, after each class, the students will be asked to complete a peer assessment (only for the peers in their group) in order to provide feedback and promote a peer-to-peer learning environment and accountability for preparing prior to classes [5,36].

## 3. Discussion

Flipping the dental anatomy classroom promises to revolutionize the teaching of tooth morphology in dental school, by articulating cognitive and psychomotor learning, and facilitating the transition to patient care in a clinical scenario [36,37]. The flipped classroom model offers several advantages compared with the traditional lecture model in dental anatomy. First, it promotes a student-centered environment, allowing students to assume responsibility for their own learning. In this model, the instructor will act as a facilitator for student learning instead of providing information to them directly [3,36]. Second, incorporating digital resources provide students with unlimited access to instructional materials. In addition, these resources stimulate interactivity and independence in the learning experience, resulting in an active instructional process [16,21]. Third, offers a more efficient use of the face-to-face time by delivering the basic content online, outside of the classroom. This will allow the instructor to create tailored activities to promote critical thinking and a TBL environment. Likewise, this allows for the incorporation of a simulation laboratory, making exercises more meaningful and allowing better integration with the rest of the pre-patient and patient-care curricula. Moreover, the exercises will help to develop psychomotor learning, including visual skills (acquiring visual discrimination), and motor skills (to recreate normal tooth morphology and restore clinical preparations). Fourth, the flipped classroom will offer dental students the opportunity to assess their own work, identifying their weaknesses and opportunities for improvement by receiving timely feedback from peers and instructors [12,21,36].

Although the flipped classroom offers many advantages, several challenges can be expected by both, students and instructors. One of the main challenges is the heavy reliance on student self-motivation and self-discipline to prepare before each face-to-face session. A strategy to minimize this limitation is incorporating a low stakes quiz prior the class to guarantee students compliance. Likewise, concerns have been raised regarding the increase in workload and preparation time for the student. Therefore, careful consideration of student’s workloads needs to be undertaken, balancing the pre-class material, and establishing clear learning goals and desired results [38,39,40,41]. From the instructor side, some challenges can be also expected. The role of the instructor is important as a facilitator and to provide feedback. Instructors unfamiliar with the flipped classroom methodology might play a passive role during the class, resulting in poor feedback and lack of reinforcement of the small-groups discussion. Likewise, flipping the dental anatomy classroom will demand more preparation time (at least for the initial implementation), creation of multiple suitable problem scenarios/quiz/clinical cases, and audiovisual content. Application of this model will require a change in educational philosophy, especially among faculties who mostly lecture. Additionally, institutions might be reluctant to include these models due to the need for more instructors, staff development, and support. To overcome these limitations, it is imperative to train the instructors in flipped classroom methodology. Additionally, the incorporation of fourth-year dental students to mentor pre-clinical labs supporting the instructor has been reported as a valid and effective solution [5,21,42].

## 4. Conclusions

The flipped classroom method has been incorporated successfully in medical and dental education. This model can enhance teaching and maximize the use of time and resources. Flipping the dental anatomy classroom is a promising method that allows students to take control of the development of their cognitive and psychomotor skills; whereas, the instructor facilitates and supports the student learning process. Further long-term studies exploring this educational model in dentistry and dental anatomy, including its implementation, students’ engagement and assessment, and strategies to overcome unresolved limitations and barriers are needed.

## References

[1] De Azevedo Rde A., da Rosa W.L., da Silva A.F., Correa M.B., Torriani M.A., Lund R.G. (2015). Comparative effectiveness of dental anatomy carving pedagogy: A systematic review. J. Dent. Educ..

[2] De Azevedo R.A., Correa M.B., Torriani M.A., Lund R.G. (2017). Optimizing quality of dental carving by preclinical dental students through anatomy theory reinforcement. Anat. Sci. Educ..

[3] Abu Eid R., Ewan K., Foley J., Oweis Y., Jayasinghe J. (2013). Self-directed study and carving tooth models for learning tooth morphology: Perceptions of students at the University of Aberdeen, Scotland. J. Dent. Educ..

[4] Lone M., McKenna J.P., Cryan J.F., Downer E.J., Toulouse A. (2018). A survey of tooth morphology teaching methods employed in the United Kingdom and Ireland. Eur. J. Dent. Educ..

[5] Obrez A., Briggs C., Buckman J., Goldstein L., Lamb C., Knight W.G. (2011). Teaching clinically relevant dental anatomy in the dental curriculum: Description and assessment of an innovative module. J. Dent. Educ..

[6] Mitov G., Dillschneider T., Abed M.R., Hohenberg G., Pospiech P. (2010). Introducing and evaluating morphodent, a web-based learning program in dental morphology. J. Dent. Educ..

[7] Al-Thobity A.M., Farooq I., Khan S.Q. (2017). Effect of software facilitated teaching on final grades of dental students in a dental morphology course. Saudi Med. J..

[8] Kieser J., Livingstone V., Meldrum A. (2008). Professional storytelling in clinical dental anatomy teaching. Anat. Sci. Educ..

[9] Jonassen D.H., Hernandez-Serrano J. (2002). Case-based reasoning and instructional design: Using stories to support problem solving. Educ. Technol. Res. Dev..

[10] Tain M., Schwartzstein R., Friedland B., Park S.E. (2017). Dental and medical students’ use and perceptions of learning resources in a human physiology course. J. Dent. Educ..

[11] Bogacki R.E., Best A., Abbey L.M. (2004). Equivalence study of a dental anatomy computer-assisted learning program. J. Dent. Educ..

[12] Nance E.T., Lanning S.K., Gunsolley J.C. (2009). Dental anatomy carving computer-assisted instruction program: An assessment of student performance and perceptions. J. Dent. Educ..

[13] Bakr M.M., Massey W., Alexander H. (2013). Evaluation of simodont^®^ haptic 3D virtual reality dental training simulator. Int. J. Dent. Clin..

[14] Bakr M.M., Massey W., Alexander H. (2014). Students’ evaluation of a 3DVR haptic device (Simodont^®^). Does early exposure to haptic feedback during preclinical dental education enhance the development of psychomotor skills?. Int. J. Dent. Clin..

[15] Roy E., Bakr M.M., George R. (2017). The need for virtual reality simulators in dental education: A review. Saudi Dent. J..

[16] Bakr M., Massey W., Alexander H. (2015). Can virtual simulators replace traditional preclinical teaching methods: A students’ perspective. Int. J. Dent. Oral Health.

[17] Bakr M.M., Massey W.L., Massa H.M. (2016). Digital cadavers: Online 2D learning resources enhance student learning in practical head and neck anatomy within dental programs. Educ. Res. Int..

[18] Jambi S., Khalifah A.M., Fadel H.T. (2015). Shifting from traditional lecturing to interactive learning in Saudi dental schools: How important is staff development?. J. Taibah Univ. Med. Sci..

[19] Lam M.T., Kwon S.R., Qian F., Denehy G.E. (2015). Evaluation of an Innovative Digital Assessment Tool in Dental Anatomy. J. Contemp. Dent. Pract..

[20] Kwon S.R., Hernandez M., Blanchette D.R., Lam M.T., Gratton D.G., Aquilino S.A. (2015). Effect of computer-assisted learning on students’ dental anatomy waxing performance. J. Dent. Educ..

[21] McLaughlin J.E., Roth M.T., Glatt D.M., Gharkholonarehe N., Davidson C.A., Griffin L.M., Esserman D.A., Mumper R.J. (2014). The flipped classroom: A course redesign to foster learning and engagement in a health professions school. Acad. Med..

[22] Nishigawa K., Omoto K., Hayama R., Okura K., Tajima T., Suzuki Y., Hosoki M., Shigemoto S., Ueda M., Rodis O.M.M. (2017). Comparison between flipped classroom and team-based learning in fixed prosthodontic education. J. Prosthodont. Res..

[23] Shin J., Brock T.P. (2017). Content delivery models influence class preparation, study habits, and preferences. Pharm. Educ..

[24] Prober C.G., Khan S. (2013). Medical education reimagined: A call to action. Acad. Med..

[25] Khan S. (2012). The One World Schoolhouse: Education Reimagined.

[26] Crothers A.J., Bagg J., McKerlie R. (2017). The flipped classroom for pre-clinical dental skills teaching—A reflective commentary. Br. Dent. J..

[27] Bohaty B.S., Redford G.J., Gadbury-Amyot C.C. (2016). Flipping the classroom: Assessment of strategies to promote student-centered, self-directed learning in a dental school course in pediatric dentistry. J. Dent. Educ..

[28] Faraone K.L., Garrett P.H., Romberg E. (2013). A blended learning approach to teaching pre-clinical complete denture prosthodontics. Eur. J. Dent. Educ..

[29] Gadbury-Amyot C.C., Redford G.J., Bohaty B.S. (2017). Dental students’ study habits in flipped/blended classrooms and their association with active learning practices. J. Dent. Educ..

[30] Aly M., Elen J., Willems G. (2004). Instructional multimedia program versus standard lecture: A comparison of two methods for teaching the undergraduate orthodontic curriculum. Eur. J. Dent. Educ..

[31] Eachempati P., Kumar K.S.K., Ismail A.R.H. (2018). The Flipped Classroom in Dental Education-Learning beyond the Four Walls of the Classroom.

[32] Zain-Alabdeen E.H. (2017). Perspectives of undergraduate oral radiology students on flipped classroom learning. Perspectives.

[33] Druzinsky R.E., Doubleday A.F. (2017). Flipping the anatomy and neuroanatomy classrooms in the first year of dental school. FASEB J..

[34] Ihm J., Choi H., Roh S. (2017). Flipped-learning course design and evaluation through student self-assessment in a predental science class. Korean J. Med. Educ..

[35] Chutinan S., Riedy C.A., Park S.E. (2017). Student performance in a flipped classroom dental anatomy course. Eur. J. Dent. Educ..

[36] Park S.E., Howell T.H. (2015). Implementation of a flipped classroom educational model in a predoctoral dental course. J. Dent. Educ..

[37] Bakr M.M., Massey W.L., Massa H.M. (2016). Flipping a dental anatomy course: A retrospective study over four years. Educ. Res. Int..

[38] Chen F., Lui A.M., Martinelli S.M. (2017). A systematic review of the effectiveness of flipped classrooms in medical education. Med. Educ..

[39] Saunders A., Green R., Cross M. (2017). Making the most of person-centred education by integrating flipped and simulated teaching: An exploratory study. Nurse Educ. Pract..

[40] Houssein S., Di Marco L., Schwebel C., Luengo V., Morand P., Gillois P. (2018). Consequences of switching to blended learning: The Grenoble medical school key elements. Stud. Health Technol. Inform..

[41] Khanova J., Roth M.T., Rodgers J.E., McLaughlin J.E. (2015). Student experiences across multiple flipped courses in a single curriculum. Med. Educ..

[42] Schlairet M.C., Green R., Benton M.J. (2014). The flipped classroom: Strategies for an undergraduate nursing course. Nurse Educ..

